# Phytochemical Optimization and Anti-Inflammatory Mechanism of an Aerial-Part Extract from *Echinacea purpurea* in DSS-Induced Colitis

**DOI:** 10.3390/ph19010109

**Published:** 2026-01-07

**Authors:** Huanhuan Jia, Geng Lu, Sa Huang, Chuangzan Yang, Zhixuan Peng, Junfeng Ban, Huanling Xing, Hong Wu

**Affiliations:** 1 Guangdong Technology Research Center for Traditional Chinese Veterinary Medicine and Natural Medicine, South China Agricultural University, Guangzhou 510006, China; 2 Medical Devices Research & Testing Center, South China University of Technology, Guangzhou 510006, China; 3 The Innovation Team for Integrating Pharmacy with Entrepreneurship, Guangdong Pharmaceutical University, Guangzhou 510006, China; 4 Department of Pharmacy, Luohu Hospital of Traditional Chinese Medicine, Shenzhen 518001, China; 5 Guangdong Provincial Key Laboratory of Advanced Drug Delivery Systems, Guangdong Pharmaceutical University, Guangzhou 510006, China

**Keywords:** *Echinacea purpurea*, spectrum-effect relationship, intestinal absorption, sustainable development and utilization

## Abstract

**Objective**: *Echinacea purpurea*, an herb with diverse pharmacological activities, has its roots widely used for anti-inflammatory and immunomodulatory purposes. Interestingly, its aerial parts, which are also rich in bioactive compounds, remain underutilized. This study aims to optimize the extraction and purification processes to obtain the aerial part extract of *Echinacea purpurea* (APE-EP) to enhance the content of active constituents and improve its anti-inflammatory and immunomodulatory effects. **Methods**: We analyzed the chemical composition of APE-EP using HPLC-MS. The intestinal absorption characteristics of APE-EP were evaluated using an ex vivo everted gut sac assay. Furthermore, the anti-inflammatory and immunomodulatory effects of APE-EP were validated using a DSS-induced colitis mouse model. **Results**: Several phenolic acids were identified, including chicoric acid and caffeic acid, which have significant antioxidant and anti-inflammatory activities. The everted gut sac assay revealed concentration-dependent absorption of chicoric acid in the gut. Results from the mouse model showed that APE-EP promoted macrophage polarization from pro-inflammatory M1 to anti-inflammatory M2 macrophages at the lesion sites, effectively suppressing inflammation and alleviating colitis-related pathological damage. **Conclusions**: This study enhances the medicinal value of the *E. purpurea*, provides new insights for the efficient utilization of plant resources, and offers a potential natural drug candidate for inflammatory bowel disease treatment.

## 1. Introduction

Ulcerative colitis (UC) is a chronic inflammatory disease characterized by damage to the colonic epithelial barrier and disruption of inflammatory homeostasis, leading to symptoms such as abdominal pain, diarrhea, bloody stools, and weight loss. Chronic damage may increase the risk of cancer [1,2]. Additionally, the enteric nervous system can be affected, potentially causing symptoms such as depression [3]. The pathogenesis of UC remains unclear, but it is believed to be associated with oxidative damage, inflammation, and disruption of the intestinal barrier. Current clinical treatments for UC focus on relieving symptoms, reducing inflammation, and modulating immune function [4].

Herbal medicine is a popular approach for disease prevention and treatment. However, the complex bioactive components of herbs pose challenges for their research and utilization. *Echinacea purpurea* (L.) Moench, a member of the Asteraceae family, has been used medicinally since the 18th century for treating influenza, wound healing, and immune deficiency. It contains various bioactive compounds, including polysaccharides, glycoproteins, alkylamides, and caffeic acid derivatives, which exhibit antioxidant, anti-inflammatory, immunomodulatory, and antimicrobial properties [5]. The roots of *E. purpurea* are the primary medicinal parts, rich in caffeic acid derivatives and thus possess strong anti-inflammatory effects. However, cultivation of *E. purpurea* is time-consuming and costly. Therefore, efficient utilization of plant resources is essential to enhance its practical value. Recently, researchers have found that parts not conventionally used for medicinal purposes, such as stems, leaves, and flowers, also contain similar bioactive compounds, providing a new direction for its medicinal research [6,7].

Previous studies have successfully identified bioactive components in the aerial parts. For instance, Classen et al. [8] isolated an arabinogalactan-protein from the pressed juice of the aerial parts, characterized by arabinose and glucuronic acid units at the glycosyl terminals. Furthermore, polysaccharides isolated from the aerial parts, specifically inulin-type fractions and acidic highly branched arabinogalactans, have been reported to possess immunostimulatory properties [9]. More recently, three polysaccharide fractions (EPPS 1, EPPS 2, and EPPS 3) were isolated from *E. purpurea* using DEAE ion exchange and gel filtration chromatography, demonstrating that EPPS 3 exhibited significant anti-inflammatory effects compared to the other fractions [10]. These findings suggest that the aerial parts are a viable and sustainable source of pharmacological agents. There is a paucity of data regarding the systematic optimization of extraction processes to maximize the overall yield of active constituents. Purification processes, as an important means of modern herbal research, can further refine the bioactive components of *E. purpurea*, improving its therapeutic efficacy, reducing impurities, and minimizing resource consumption and costs in practical applications. This study aims to optimize the extraction process to increase the content of active constituents in the aerial part extract of *E. purpurea* (APE-EP) and enhance its anti-inflammatory and immunomodulatory effects. We systematically analyzed the chemical composition of APE-EP using HPLC-MS, revealing its complex chemical profile. Additionally, we evaluated the intestinal absorption characteristics of APE-EP using an ex vivo everted gut sac assay and validated its anti-inflammatory and immunomodulatory effects using a DSS-induced colitis mouse model. Immunohistochemistry and immunofluorescence analyses showed that APE-EP promotes the polarization of macrophages at the lesion sites from pro-inflammatory M1 macrophages to anti-inflammatory M2 macrophages, thereby effectively suppressing inflammation and alleviating colitis pathological damage. These findings provide scientific evidence for the efficient utilization of the aerial parts of *E. purpurea* and offer a potential candidate for the development of new natural drugs.

## 2. Results

### 2.1. Optimization of Multi-Indicator Extraction Process

**Figure 1:**
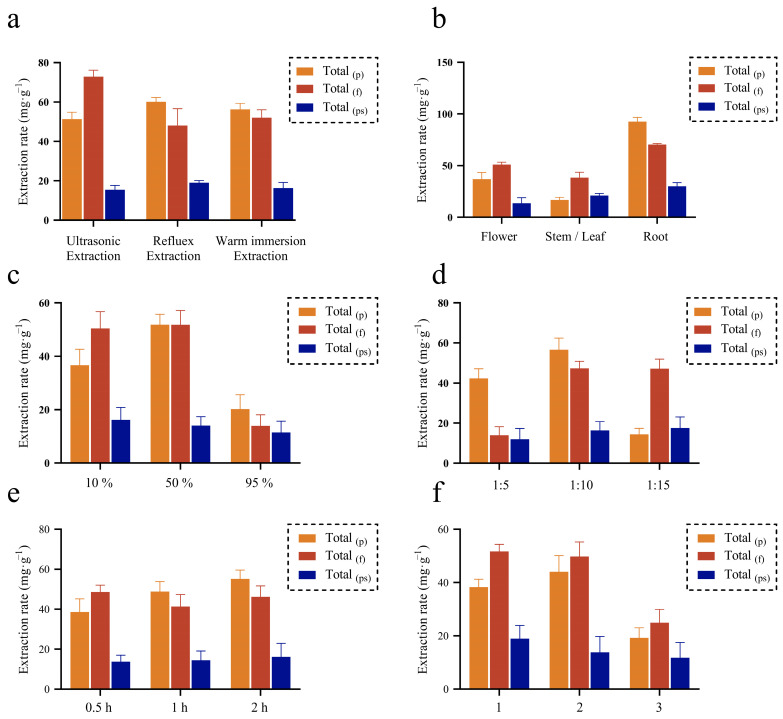
Effect of different factors on the extraction rate of multi-components. (**a**): Extraction method; (**b**): Medicinal part; (**c**): Alcohol concentration; (**d**): Ratio of feed to liquid; (**e**): Extraction time; (**f**): The number of extraction times at a fixed time; Total (p) is the content of total polyphenols; Total (f) is the content of total flavonoids; Total (ps) is the content of total polysaccharides.

The extraction process affects the composition and content of active components in the aerial part extract of *E. purpurea* (APE-EP), thereby determining its pharmacological activity. The experimental investigation focused on extraction methods, solvents, extraction time, and the number of extraction cycles. Figure 1a examines the impact of three extraction methods—ultrasonic extraction, reflux extraction, and conventional solvent extraction—on the components [11]. Reflux extraction yielded the highest content of total phenolics and polysaccharides in *E. purpurea*, likely because constant temperature facilitates the dissolution of active components. In contrast, flavonoids, which dissolve poorly, benefit more from energy-assisted extraction, showing better extraction efficiency with ultrasonic extraction. Figure 1b reveals differences in the content of components among different parts of *E. purpurea*. The aerial parts show similar levels of total flavonoids and polysaccharides, while the traditionally medicinal roots contain higher levels of total phenolics and flavonoids, consistent with previous studies [12]. However, when the aerial parts are utilized intensively, their extraction capability is comparable to that of the roots, suggesting that the aerial parts may possess similar pharmacological properties. Figure 1c,d analyze the effects of ethanol concentration and solid–liquid ratio. An optimal solvent polarity and solid–liquid ratio enhance the extraction of total flavonoids. However, excessively high ethanol concentration or solid–liquid ratio may increase impurities and degrade active components, thereby reducing extraction efficiency. Similarly, Figure 1e shows that prolonged extraction time can damage the structure of active components and affect extraction outcomes. Figure 1f investigates the number of extraction cycles. With a constant total extraction time, three extraction cycles result in lower total extraction efficiency due to shorter extraction duration and insufficient temperature and time for each cycle. In contrast, two extraction cycles achieve a balance between time and energy efficiency, yielding the most efficient extraction.

To further optimize the extraction conditions for APE-EP, the effects of extraction time, ethanol concentration, and solid–liquid ratio on the extraction yields of multiple components in APE-EP were examined. The specific extraction conditions and corresponding results are presented in Table 1. Using SPSS 26.0 software for weight analysis of the five indicators, total phenolics content was identified as the most influential parameter, with a weight of 47.08%. Total flavonoids content had a weight of 22.82%, total polysaccharides content had a weight of 17.35%, extract yield had a weight of 10.09%, and DPPH scavenging rate had a weight of 2.68%.

Based on the weighted scores, the optimized extraction process for APE-EP was determined to be as follows: ethanol concentration of 50%, solid–liquid ratio of 1:10, and hot maceration at 80 °C for two cycles of 0.5 h each. Under these conditions, the total phenolic content was measured to be 72.18 ± 1.31 μg·mL^−1^ (RSD ≤ 3%).

### 2.2. Characterization of the Chemical Constituents of APE-EP

**Figure 2:**
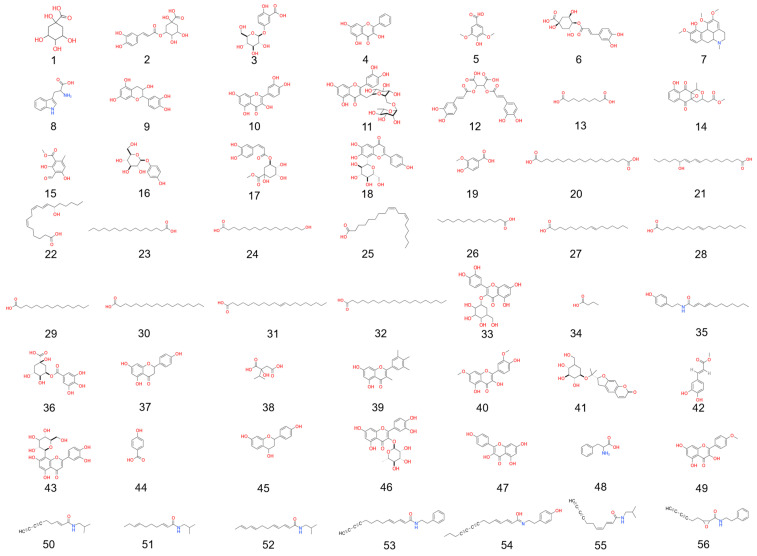
Structural formula of identified chemical components from APE-EP.

To further identify the active components in APE-EP, high-performance liquid chromatography–mass spectrometry (HPLC-MS) was employed, combined with modern instrumental methods for analyzing the “structure–activity relationship” of the components. A total of 56 active components were identified in APE-EP (Figure 2, Supplementary Figure S2 and Table S10), including 15 flavonoids, 13 organic acids, 13 fatty acids, 8 n-alkylamides, and 2 amino acids, as well as alkaloids, phenylpropanoids, and glycosides. Among these, phenolic acids such as quinic acid (1), chlorogenic acid (6), chicoric acid (12), and vanillic acid (19) are known for their strong antioxidant, anti-inflammatory, and immunomodulatory activities. During the extraction process, phenolic acids were selectively enriched as major components, and the contents of selected phenolic acids were determined to evaluate the anti-inflammatory and immunomodulatory potential of APE-EP. Using caffeic acid as the internal standard, the contents of caffeic acid, chlorogenic acid, and chicoric acid in APE-EP were measured to be 1.54–1.68%, 0.11–0.16%, and 1.99–2.20%, respectively. These levels are higher than those reported for extracts from other parts of *E. purpurea*. Therefore, the enhanced extraction and purification process thus increased the content of these bioactive compounds [6], suggesting that APE-EP has potential anti-inflammatory and immunomodulatory activities [13,14].

### 2.3. Characterization of the Intestinal Absorption of APE-EP

The absorption and utilization of active components are critical for their pharmacological efficacy. To investigate the potential of APE-EP for treating UC, the intestinal absorption characteristics of chicoric acid were evaluated using an ex vivo everted gut sac model. Table 2 presents the cumulative absorption (*Q*) of chicoric acid across different intestinal segments. Contrary to linear kinetics, the cumulative absorption amount remained relatively constant despite a 10-fold increase in donor concentration (0.05 to 0.5 μg·mL^−1^) This indicates that the absorption of chicoric acid follows saturation kinetics (capacity-limited absorption) within the tested range, suggesting that the intestinal transport mechanisms are easily saturated. Furthermore, no statistically significant difference in absorption was observed among the duodenum, jejunum, ileum, and colon (*p* > 0.05). This limited intestinal permeability is therapeutically advantageous for ulcerative colitis, as it implies that the majority of the administered chicoric acid is retained within the intestinal lumen, thereby ensuring a high local concentration at the site of inflammation (the colon) to exert direct pharmacological effects.

### 2.4. Anti-Colorectal Inflammation Effect of APE-EP

**Figure 3:**
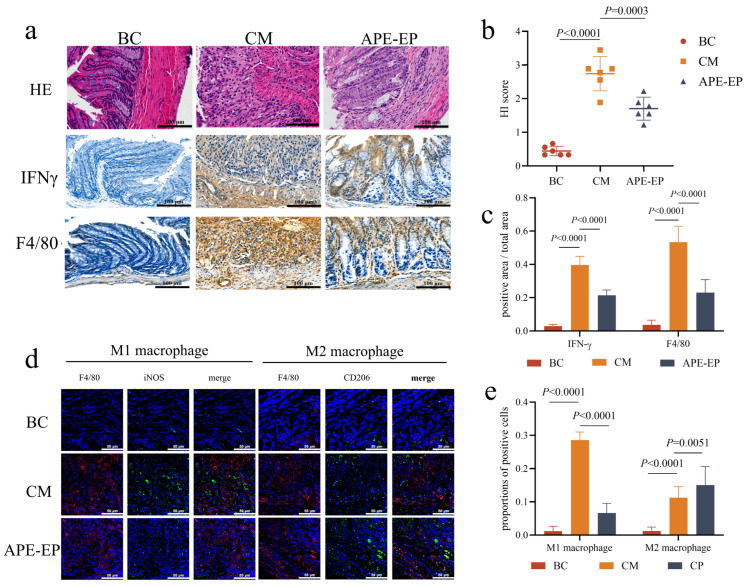
Anti-inflammatory effect of APE-EP. ((**a**): H&E staining and immunohistochemistry; (**b**): HI score of H&E staining; (**c**): Positive area of immunohistochemistry; (**d**): immunofluorescence; (**e**): Proportion of different macrophages).

The ex vivo intestinal absorption experiment reflects the intestinal absorption characteristics of the active components of APE-EP. To validate the functionality of APE-EP and explore its anti-inflammatory and immunomodulatory effects on UC, a DSS-induced colitis mouse model was used to evaluate the therapeutic potential of APE-EP. Under DSS induction, mice in the colitis model group (CM group) exhibited symptoms of UC, such as bloody stools and anal redness and swelling, while the APE-EP group showed a certain degree of alleviation of these symptoms. Compared to the blank control group (BC group), both normal mice, the CM group revealed the disappearance of intestinal cell structures, disorganized cell arrangement, intestinal lumen dilation, mucosal thickening, and significant loss of the mucus layer and crypt structures (Figure 3a,b). In contrast, APE-EP treatment (APE-EP group) reduced the extent of intestinal mucosal damage, with partial disappearance of crypt structures, appearance of twisted and atrophic states, and cellular debris within the glands, leading to a reduction in tissue damage scores (*p* = 0.0003). Immunohistochemistry analyzed the expression of the pro-inflammatory cytokine IFN-γ and the distribution of macrophages in the inflamed regions [15]. IFN-γ, a pro-inflammatory cytokine secreted by immune cells, serves as an important recruiter and activator of macrophages, neutrophils, and NK cells, regulating immune responses and participating in inflammatory processes. Under DSS induction, IFN-γ expression was significantly upregulated (*p* < 0.0001), whereas APE-EP treatment markedly downregulated IFN-γ secretion in the intestinal regions (*p* < 0.0001), indicating that APE-EP can alleviate inflammatory responses at the lesion sites. Similarly, F4/80, a specific marker for macrophages, showed comparable changes in immunohistochemical staining, with APE-EP reducing the distribution of macrophages at the lesion sites (*p* < 0.0001) (Figure 3c). Immunofluorescence further investigated macrophage polarization, using iNOS and CD206 to label M1 and M2 macrophages, respectively (Figure 3d,e). The results demonstrated that APE-EP significantly suppressed the number of pro-inflammatory M1 macrophages (*p* < 0.0001) and upregulated the number of anti-inflammatory M2 macrophages (*p* = 0.0051). APE-EP may induce the polarization of M1 macrophages toward M2 macrophages, thereby modulating immune responses and achieving anti-inflammatory effects.

## 3. Discussion

Conventional development of the medicinal value of *E. purpurea* has focused on its roots; however, factors such as long growth cycles and high costs limit its large-scale application. To address this challenge, the present study focused on the abundant and renewable aerial parts, which have previously been reported as a rich source of bioactive phenolic acids [16]. We established an optimized extraction process that achieved efficient enrichment of these phenolic compounds, a class of molecules fundamentally linked to the plant’s antioxidant and therapeutic potential [17]. Standardization is critical, as early studies noted that inconsistent extraction could lead to poor correlation between marker components and anti-inflammatory activity [18]. This optimized protocol not only provides technical support for the high-value utilization of *E*.’s aerial parts but also furnished a standardized, component-rich extract (APE-EP), constituting the material premise for the subsequent pharmacological investigation.

The in vivo efficacy of an oral formulation is contingent upon the presence of its active components at the target tissue. Previous research have indicated that caffeic acid derivatives, such as chicoric acid, often undergo rapid metabolism and exhibit limited systemic bioavailability [19]. Consistent with this report, our ex vivo everted gut sac results showed that the overall cumulative absorption of active components was relatively low. However, crucially, we observed that the representative component, chicoric acid, exhibits the highest absorption rate constant in the colon compared to other segments. This specific pharmacokinetic behavior presents a therapeutic advantage for ulcerative colitis: the limited systemic absorption implies that a high concentration of active components is retained in the intestinal lumen, allowing for direct and effective exposure at the site of inflammation (the colon). This elucidates the specific pharmacokinetic mechanism, extending recent findings that focused primarily on general phytochemical profiling [20]. This provides a pharmacokinetic rationale for the significant therapeutic efficacy observed in the animal model.

Building upon the confirmed colonic bioavailability of the active components, this study further elucidated the cellular mechanism of action of APE-EP in a DSS-induced colitis model. In the pathogenesis of colitis, inflammatory mediators such as IFN-γ are known to drive the polarization of macrophages towards a pro-inflammatory M1 phenotype, which exacerbates tissue damage [21]. Our data reveal that APE-EP’s core therapeutic action is the precise modulation of this process. APE-EP intervention effectively reversed the pathological macrophage polarization by significantly inhibiting the enrichment of the pro-inflammatory M1 phenotype (iNOS+) while promoting the generation of the anti-inflammatory and tissue-reparative M2 phenotype [22,23] which aligns with recent evidence supporting chicoric acid’s therapeutic role in regulating intestinal inflammation. This remodeling of the macrophage subset balance is the core mechanism behind APE-EP’s ability to suppress local inflammation. This effect is mechanistically plausible, as the phenolic acids enriched in APE-EP are known to possess potent immunomodulatory properties [24]. Specifically, chicoric acid has been demonstrated to suppress M1 macrophage activation by directly inhibiting the TLR4/NF-κB signaling pathway [25,26],which aligns with recent evidence supporting chicoric acid’s therapeutic role in regulating intestinal inflammation [27]. Thus, we establish a direct link from the extract’s key chemical constituents to its cellular function and, ultimately, its overall therapeutic efficacy. It is worth noting that only a single dose (250 mg/kg) was employed in the reported experiments. Although this dose was sufficient to validate the therapeutic potential of the formulation, further research is warranted to establish a detailed dose–response curve and identify the optimal dosage for maximizing efficacy while minimizing potential side effects.

In conclusion, this study not only provides systematic process and pharmacological data for the effective utilization of E.’s aerial parts but also reveals a complete pathway of action—from chemical composition and intestinal absorption to cellular target—by integrating multi-dimensional evidence. This integrative research strategy offers a paradigm for elucidating the material basis of efficacy and the mechanisms of action for other complex natural products. Future research could focus on isolating and identifying the key molecular entities within APE-EP that govern macrophage polarization and evaluating their therapeutic potential in a broader range of chronic inflammatory disease models.

## 4. Materials and Methods

### 4.1. Materials

Analytical Standard including caffeic acid, chlorogenic acid and chicoric acid were purchased from Shanghai Standard Technology Co., Ltd. (Shanghai, China). Acetonitrile, methanol and other chromatographic pure reagents and mass spectrometry grade reagents were purchased from Thermo Fisher Scientific. (Waltham, MA, USA). Phosphate buffer (PBS), sodium citrate antigen repair solution, goat closed serum, endogenous peroxidase blocker and other reagents were purchased from Wuhan Boster Biological Technology Co., Ltd. (Wuhan, China). Neutral resin, progressive rapid hematoxylin staining solution, etc., were purchased from Guangzhou Kaixiu Trading Co., Ltd. (Guangzhou, China).

### 4.2. Preparation and Purification of APE-EP

The *E. purpurea* seeds (PI 633670) used in this study were obtained from the USA Seed Bank. The plants were cultivated in the Qilin Experimental Field at South China Agricultural University and harvested at full bloom after 16 months. A voucher specimen of the aerial parts was prepared and authenticated by Prof. Hong Wu. This specimen has been deposited in the Herbarium of the Guangdong Technology Research Center for Traditional Chinese Veterinary Medicine and Natural Medicine, South China Agricultural University, under the voucher number [EP-AP-2020-01]. The entire plants were air-dried naturally, cut into segments of 2–4 cm, and stored in a cool repository for extraction and subsequent experiments. To simulate practical industrial processing conditions and maximize resource efficiency, the stems and leaves were harvested and processed together as the aerial parts. This approach was adopted because separating stems from leaves on a large scale is labor-intensive and increases production costs. To maximize the yield of active constituents, the extraction parameters (extraction time, ethanol concentration, and solid–liquid ratio) were systematically optimized using an L9(3^4^) orthogonal array design (detailed optimization data are provided in Supplementary Material, Tables S1–S5). Based on the optimized conditions, The preparation method for the *E. purpurea* extract was as follows: The aerial parts of *E. purpurea* were cut into pieces less than 4 cm in length and mixed with 10 volumes of 50% ethanol. The mixture was subjected to two rounds of hot maceration at (80 ± 1) °C for 1 h each, followed by filtration and collection of the filtrate. The filtrate was then concentrated under reduced pressure at (60 ± 1) °C to a relative density of 1.15–1.18. Subsequently, to enhance purity, the concentrated extract was subjected to a coupled resin chromatography purification process. First, the extract was diluted to 1 mg·mL^−1^, adjusted to pH 2.0, and passed through a column packed with D900 macroporous adsorption resin at a flow rate of 1.0 mL·min^−1^. After adsorption equilibrium, the target fraction was eluted with 70% ethanol. The eluate was subsequently adjusted to pH 4.0 and further purified using a polyamide resin column, eluting with 70% ethanol. Finally, the eluate was concentrated and spray-dried to obtain the purified APE-EP powder.

### 4.3. Characterization of the Chemical Constituents of APE-EP

#### 4.3.1. Determination of Active Ingredient Content

The total phenols, flavonoids, and polysaccharides in the extract were determined using the Folin–Ciocalteu method [28], the sodium nitrite–aluminum nitrate–sodium hydroxide method [29], and the phenol–sulfuric acid method [30], respectively. Each method utilized UV–Vis spectrophotometry to measure absorbance and accurately calculate the concentrations of the respective components in the test solutions. Additionally, to evaluate the antioxidant capacity of the extract, its scavenging efficiency against DPPH radicals was determined using the method described by Lin [17].

#### 4.3.2. Identification and Quantitative Analysis of Active Substances

In this study, the chemical profile of APE-EP was characterized using a Shimadzu Nexera UHPLC LC-30A system (Shimadzu, Kyoto, Japan) coupled with a high-resolution AB SCIEX TripleTOF 5600+ mass spectrometer (SCIEX, Framingham, MA, USA). The analysis was conducted using electrospray ionization (ESI) in both positive and negative ion modes. Specific source conditions, including gas flow rate, source temperature, and voltage, were optimized to enhance detection efficiency [31]. The chromatographic separation was achieved using a Waters BEH C18 column (Waters Corporation, Milford, MA, USA) with a mobile phase consisting of acetonitrile and 0.1% formic acid, employing gradient elution for efficient component separation. The acquired MS data were converted using the Analysis Base File Converter software(Reifycs Inc., Tokyo, Japan) and processed with MS-DIAL software(RIKEN, Yokohama, Japan; Version 4.70) to generate a data matrix in CSV format. Additionally, the MS1 and MS2 data were comprehensively analyzed by comparing the mass spectral fragmentation patterns, relative retention times, and cleavage pathways with those reported in the literature, using databases such as MassBank, Respect, and GNPS to identify and confirm the structures of the compounds. Finally, the analyzed compounds were plotted by KingDraw V5.0.0 software (Qingdao KingDraw Information Technology Co., Ltd., Qingdao, China).

For quantitative analysis, the contents of three marker components (chicoric acid, chlorogenic acid, and caffeic acid) were determined using HPLC with UV detection at 332 nm based on the Quantitative Analysis of Multi-Components by Single Marker (QAMS) method. Caffeic acid was selected as the internal reference substance to calculate the contents of other components using their relative correction factors (RCFs). The detailed calculation procedures for the QAMS method, including the equations for relative correction factors (f_s/i_) and the evaluation of method robustness, are described in Section S3 of the Supplementary Materials. The chromatographic separation for both analyses was achieved using a Waters BEH C18 column with a mobile phase consisting of acetonitrile and 0.1% formic acid under gradient elution. Detailed validation data are provided in the Supplementary Material (Figure S1 and Tables S6–S8).

### 4.4. Evaluation of Intestinal Absorption Properties of APE-EP

In this study, we constructed an ex vivo everted gut sac model to evaluate the oral absorption capacity of APE-EP, using chicoric acid—an anti-inflammatory compound in APE-EP—as the indicator [32]. The concentration of absorbed chicoric acid was determined by HPLC. The experimental procedure was as follows: 12 Male SD rats were fasted for 24 h with free access to water. Under sodium pentobarbital anesthesia, a midline laparotomy was performed to create intestinal sacs in the duodenum and colon. The sacs were rinsed with pre-warmed physiological saline, cannulated, and washed with K-R solution at (37 ± 0.5) °C to remove mesentery and fat. The intestinal segments were rotated and ligated into small sacs of 10 cm each, which were then equilibrated with blank K-R solution for 15 min. The intestinal sacs were incubated in K-R solution containing different concentrations of APE-EP (0.05, 0.1, and 0.5 μg·mL^−1^) at 37 °C for 15 min. Every 15 min, 0.25 mL of the perfusate was collected and replaced with an equal volume of K-R solution for 150 min. The collected perfusates were filtered through a 0.22 µm membrane and analyzed by HPLC to determine the concentration of chicoric acid. At the end of the experiment, the length and circumference of each intestinal segment were measured to calculate the surface area, which was used to derive the cumulative absorption amount (Q) of chicoric acid.

The research protocols were meticulously reviewed and granted approval by the Institutional Animal Care and Use Committee (IACUC) at Guangdong Pharmaceutical University (GDPULAC2021031).

(1) $$Q=(C_{n}\times 2+\sum_{i=1}^{n-1}C_{i}\times 0.25)/A,$$

where C_n_ indicates the chicoric acid concentration at time point t, μg·mL^−1^; Ci indicates the chicoric acid concentration at the time point before t time point, μg·mL^−1^; A indicates the area of the intestinal segments.

### 4.5. Anti-Colorectal Inflammation Effect Study

To investigate the immunomodulatory and anti-inflammatory activities of APE-EP, a dextran sodium sulfate (DSS)-induced colitis mouse model was established and treated with the extract [33]. 18 male C57BL/6 mice, procured from the Guangdong Medical Laboratory Animal Center, were housed in SPF facility with a temperature of 22 ± 2 °C, relative humidity of 50–60%, and a 12 h light/dark cycle. All mice had ad libitum access to standard chow and sterile water. After acclimated for 7 days and then randomly divided into control, model, and treatment groups based on body weight. From day 8 to 14, all groups except the control group received 3% DSS in drinking water to induce colitis. Based on our preliminary acute toxicity assessment, the Maximum Tolerated Dose (MTD) of APE-EP in mice was determined to be 250.20 mg·kg^−1^. Consequently, a therapeutic dosage of 250 mg·kg^−1^ (approximating the MTD) was selected for this study to evaluate the maximal potential efficacy of the extract within the safety margin. Mice in the treatment group were administered 250 mg·kg^−1^ of the extract via gavage daily, while the control and model groups received an equal volume of saline for 15 consecutive days. During the experiment, the body weight, fur condition, and overall health of the mice were closely monitored and recorded. At the end of the experiment, the mice were anesthetized with sodium pentobarbital and euthanized by cervical dislocation. The distal colon tissues were then excised and fixed in 10% neutral formalin for subsequent histological evaluation. The research protocols were meticulously reviewed and granted approval by the Institutional Animal Care and Use Committee (IACUC) at Guangdong Pharmaceutical University (GDPULAC2021031).

#### 4.5.1. Histological Analysis

In this study, the colorectal tissues were dehydrated, paraffin-embedded, and sectioned into 4 μm slices, followed by hematoxylin and eosin (H&E) staining. Tissue sections were observed the histomorphometric changes by light microscopy and evaluated for the degree of inflammation based on established scoring criteria [4,34].

#### 4.5.2. Immunohistochemical Analysis

Immunohistochemical (IHC) analysis of macrophage markers (IFN-γ and F4/80) was performed on colorectal paraffin sections. Sections were deparaffinized and subjected to antigen retrieval in sodium citrate buffer under high pressure for 2 min. After cooling to room temperature and washing with PBS, sections were incubated in 3% hydrogen peroxide for 10 min to block endogenous peroxidase activity. Sections were then blocked with goat serum for 1 h, followed by incubation with primary antibodies against IFN-γ and F4/80 (both diluted 1:300) overnight at 4 °C. On the next day, sections were warmed for 30 min, washed with PBS to remove unbound primary antibodies, and incubated with secondary antibodies for 1 h at room temperature. Sections were stained with DAB chromogen for 1 min and the reaction was quenched with water. Sections were counterstained with hematoxylin for 35 s, followed by washing to remove excess stain. All sections were observed and photographed under an optical microscope (DM300LED), and the positive expression areas were quantified using ImageJ software (National Institutes of Health Bethesda, MD, USA; Version 1.53c).

#### 4.5.3. Immunofluorescence Analysis

In this study, immunofluorescence was used to label and analyze M1 and M2 macrophages in colorectal tissue sections. Sections were deparaffinized and rehydrated, then washed twice in 0.5% PBST to remove residual wax. Subsequently, sections were immersed in 0.5% Triton X-100 solution for 10 min to increase cell membrane permeability. After washing, sections were blocked with 10% goat serum at room temperature for 1 h to reduce nonspecific binding. A mixture of primary antibodies was added to the sections and incubated overnight at 4 °C for specific binding to target antigens. Following incubation, the primary antibody dilution was removed, and sections were washed with PBST before incubation with a mixture of secondary antibodies at room temperature for 1 h. After incubation, sections were washed again with PBST and PBS to remove unbound secondary antibodies. Finally, nuclei were stained with DAPI, and sections were mounted and observed using a laser confocal microscope (STELLARIS 5, Leica Microsystems, Wetzlar, Germany) to capture images. This method provides a precise assessment of the distribution and quantity of M1 and M2 macrophages in tissues, offering intuitive cellular evidence for the effects of *E. purpurea* extract on macrophage polarization in the colitis model.

### 4.6. Data Analysis and Statistics

Data were tested for normality and homogeneity of variances. If the data met the assumptions of normality and homogeneity of variances, one-way analysis of variance (ANOVA) was performed. When significant differences were detected (*p* < 0.05), Dunnett’s test was used for multiple comparisons. If the data did not meet the assumptions of normality or homogeneity of variances, the Kruskal–Wallis test was used. When significant differences were detected (*p* < 0.05), pairwise comparisons were performed using the Mann–Whitney U test.

## 5. Conclusions

By optimizing the extraction process of the aerial parts of *E. purpurea* (APE-EP), this study successfully increased the content and pharmacological activity of active components. Systematic analysis using high-performance liquid chromatography–mass spectrometry (HPLC-MS) revealed a rich chemical profile in APE-EP, particularly significant levels of phenolic acids such as chicoric acid and caffeic acid, which play important roles in antioxidant, anti-inflammatory, and immunomodulatory activities. The ex vivo everted gut sac experiment indicated that chicoric acid, a major component of APE-EP, exhibited favorable intestinal absorption characteristics with concentration-dependent absorption. Furthermore, the anti-inflammatory and immunomodulatory effects of APE-EP were validated in a DSS-induced colitis mouse model, where APE-EP significantly reduced tissue damage and regulated macrophage polarization. This study not only provides new scientific evidence for the medicinal value of the aerial parts of *E. purpurea* but also offers new insights for the efficient utilization of plant resources and sustainable development, as well as potential natural drug candidates for the treatment of inflammatory diseases such as colitis.

## Figures and Tables

**Table 1:** Determination results of samples.

No.	Time (h)	Ethanol Concentration	Feed-Liquid Ratio	Total Polyphenols (μg·mL^−1^)	Total Flavonoids (μg·mL^−1^)	Total Polysaccharides (μg·mL^−1^)	Anointing Rate (%)	DPPH Scavenging Ability (%)	Overall Score
1	0.5	0%	1:5	28.62	19.13	6.07	50.02	33.23	2.247
2	1	0%	1:10	37.30	51.22	1.08	30.11	6.12	−0.014
3	2	0%	1:15	18.40	11.68	3.08	27.02	18.02	−1.293
4	0.5	50%	1:10	47.60	28.63	2.08	26.35	52.78	2.747
5	1	50%	1:15	46.70	32.93	0.09	15.44	12.49	−1.339
6	2	50%	1:5	30.00	29.08	2.10	42.41	29.44	−0.315
7	0.5	95%	1:15	55.20	40.19	2.09	34.3	8.22	0.221
8	1	95%	1:5	15.80	20.30	1.08	37.14	16.09	−0.930
9	2	95%	1:10	17.80	12.20	7.08	22.1	17.17	−1.324

**Table 2:** Cumulative absorption per unit area of *E. purpurea* at different time points at different mass concentrations (Q, *n* = 4).

Intestine Segment	Chicoric Acid Concentration (μg·mL^−1^)	30 min	60 min	90 min	120 min	150 min
Duodenum	0.05	0.382 ± 0.951	0.417 ± 0.314	0.582 ± 0.780	0.801 ± 0.280	0.951 ± 0.551
	0.1	0.278 ± 0.524	0.298 ± 0.153	0.315 ± 0.353	0.588 ± 0.415	0.796 ± 0.333
	0.5	0.293 ± 0.973	0.550 ± 0.884	0.654 ± 0.457	0.722 ± 0.543	0.953 ± 0.950
Jejunum	0.05	0.439 ± 0.512	0.558 ± 0.489	0.678 ± 0.203	0.714 ± 0.367	0.831 ± 0.793
	0.1	0.380 ± 0.827	0.472 ± 0.432	0.571 ± 0.117	0.615 ± 0.408	0.810 ± 0.395
	0.5	0.330 ± 0.970	0.394 ± 0.431	0.443 ± 0.872	0.610 ± 0.254	0.662 ± 0.10
Ileum	0.05	0.418 ± 0.864	0.560 ± 0.215	0.623 ± 0.826	0.795 ± 0.225	0.978 ± 0.768
	0.1	0.283 ± 0.406	0.423 ± 0.791	0.633 ± 0.937	0.748 ± 0.980	0.940 ± 0.901
	0.5	0.353 ± 0.446	0.504 ± 0.110	0.645 ± 0.812	0.844 ± 0.676	0.944 ± 0.523
Colon	0.05	0.305 ± 0.788	0.492 ± 0.767	0.687 ± 0.995	0.809 ± 0.389	0.933 ± 0.733
	0.1	0.324 ± 0.846	0.491 ± 0.473	0.643 ± 0.813	0.739 ± 0.184	0.832 ± 0.277
	0.5	0.292 ± 0.855	0.356 ± 0.815	0.512 ± 0.947	0.690 ± 0.60	0.741 ± 0.136

## Data Availability

The original contributions presented in this study are included in the article/Supplementary Materials. Further inquiries can be directed to the corresponding author.

## Supplementary Materials

The following supporting information can be downloaded at: https://www.mdpi.com/article/10.3390/ph19010109/s1, Figure S1: Chromatogram of HPLC; Figure S2: Representative UPLC-Q-TOF-MS/MS total ion chromatograms (TIC) of the aerial part extract of *E. purpurea*; Table S1: Design of factors and levels of orthogonal test; Table S2: Results and range analysis of the orthogonal test; Table S3: Eigenvalues and variance contribution rates from Principal Component Analysis (PCA); Table S4: The result of principal component factors; Table S5: Analysis of Variance (ANOVA) for the orthogonal test; Table S6: Linear regression data, LOD, and LOQ of the three phenolic acids; Table S7: Intra-day and inter-day precision of the HPLC method (n = 5); Table S8: Recovery of the three phenolic acids (n = 6); Table S9: Relative correction factors of components to be measured in samples; Table S10: LC-MS Analysis of Chemical Constituents in the aerial part extract of *E. purpurea*.
