# Supplementary material for: Phytochemical Optimization and Anti-Inflammatory Mechanism of an Aerial-Part Extract from Echinacea purpurea in DSS-Induced Colitis
Source: Pharmaceuticals (Basel). 2026 Jan 7;19(1):109. doi: 10.3390/ph19010109 (PMC12845002; doi:10.3390/ph19010109)
Supplement: Supplementary file 1 [file pharmaceuticals-19-00109-s001.zip › pharmaceuticals-4037775-supplementary.pdf]

Supplementary Material

Title: Phytochemical Optimization and Anti-Inflammatory Mechanism of an Aerial-Part Extract from *Echinacea purpurea* in DSS-Induced Colitis

1 Optimization of Extraction Process

1.1 Experimental Design

To optimize the extraction process, a three-factor, three-level orthogonal array design $L9(3^4)$ was utilized. The three factors selected for optimization were extraction time (A), ethanol concentration (B), and solid-liquid ratio (C). The assignment of these factors and their respective levels is presented in Table S1.

Table S1 Design of factors and levels of orthogonal test

	Extraction time (h)	Ethanol concentration (%)	Solid-liquid ratio (g/mL)
1	0.5	0 (water)	1:5
2	1	50%	1:10
3	2	95%	1: 15

1.2 Preparation of Test Solution

Prior to analysis, the concentrated paste obtained from the orthogonal array or single-factor experiments was diluted with water to prepare a test solution with a mass concentration of 100 $mg \cdot mL^{-1}$. An appropriate aliquot of this solution was used for the subsequent colorimetric assays.

1.3 Determination of Total Polyphenols Content (TPC)

TPC was determined using the Folin–Ciocalteu colorimetric method. Briefly, the test solution was mixed with Folin–Ciocalteu reagent and 10% sodium carbonate (Na2CO3) solution. After incubation in the dark at room temperature for 2 h, the absorbance was measured at 760 nm. The total polyphenol content was calculated using gallic acid as the standard.

1.4 3. Determination of Total Flavonoids Content (TFC)

TFC was determined using the sodium nitrite-aluminum nitrate-sodium hydroxide colorimetric method . The test solution was sequentially mixed with 5% sodium nitrite (NaNO2), 10% aluminum nitrate (Al(NO3)3), and 4% sodium hydroxide (NaOH) solutions. After reacting at room temperature for 15 min, the absorbance was measured at 510 nm. The total flavonoid content was calculated using rutin as the standard.

1.5 4. Determination of Total Polysaccharides Content

The total polysaccharide content was determined using the phenol-sulfuric acid method. The test solution was mixed with 5% phenol reagent and concentrated sulfuric acid. After incubation in the dark at room temperature for 30 min, the absorbance was measured at 490 nm. The total polysaccharide content was calculated using glucose as the standard.

1.6 Determination of DPPH Radical Scavenging Activity

The antioxidant activity was evaluated using the DPPH radical scavenging assay. Briefly, 2 mL of the test solution was mixed with 2 mL of DPPH ethanol solution (2·10-4 mol·L-1). The mixture was incubated in the dark at room temperature for 30 min. The absorbance (Asample) was measured at 517 nm. The scavenging rate was calculated as follow,

$$\text{scavenging rate}=(1-A_{sample}/A_{control})\times 100\% \quad (1)$$

where $A_{control}$ is the absorbance of the DPPH solution without the extract.

1.7 Orthogonal Test Results and Analysis

An orthogonal array design L9(3^4) was employed to optimize the extraction parameters. The raw experimental data for the five evaluation indicators (total polyphenols, total flavonoids, total polysaccharides, extract yield, and DPPH scavenging activity) are presented in Table S2.

Table S2 Results and range analysis of the orthogonal test

No.	Total Polyphenols ($\mu g\cdot mL^{-1}$)	Total Flavonoids ($\mu g\cdot mL^{-1}$)	Total Polysaccharides ($\mu g\cdot mL^{-1}$)	Extract Yield (%)	DPPH Scavenging (%)
1	28.620	19.128	6.070	50.02	33.23
2	37.300	51.219	1.075	30.11	6.12
3	18.400	11.675	3.082	27.02	18.02
4	47.600	28.628	2.077	26.35	52.78
5	46.700	32.925	0.089	15.44	12.49
6	30.000	29.081	2.099	42.41	29.44
7	55.200	40.191	2.090	34.3	8.22
8	15.800	20.300	1.081	37.14	16.09
9	17.800	12.200	7.077	22.1	17.17

1.8 Weight Analysis of Extraction Indicators

To scientifically evaluate the extraction efficiency, Principal Component Analysis (PCA) was used to determine the weights of five indicators: total polyphenols, total flavonoids, total polysaccharides, extract yield, and DPPH scavenging activity.

Table S3 Eigenvalues and variance contribution rates from Principal Component Analysis (PCA)

Indicator	Eigenvalue (λ)	Variance Contribution (%)	Cumulative Contribution (%)	Weight (%)
Total Polyphenols	2.354	47.084	47.084	47.08
Total Flavonoids	1.141	22.823	69.906	22.82
Total Polysaccharides	0.866	17.326	87.233	17.35
Extract Yield	0.505	10.091	97.323	10.09
DPPH Scavenging	0.134	2.677	100	2.68

1.9 Range Analysis of Orthogonal Test

To evaluate the overall extraction efficiency, a "Comprehensive Score" for each experimental run was calculated based on the weights determined in Table S2. Specifically, the raw data were first normalized, and then the weighted sum was calculated. Range analysis R) was performed on the comprehensive scores to determine the optimal levels and the order of influence of the factors. The results are summarized in Table S4.

Table S4 The result of principal component factors

	A: Extraction Time (h)	B: Ethanol Concentration (%)	C: Solid-Liquid Ratio (g/mL)	Factor Score	Comprehensive Score
1	0.5	0	1:5	1.465	2.247
2	1	0	1:10	-0.009	-0.014
3	2	0	1:15	-0.843	-1.293
4	0.5	50	1:10	1.791	2.747
5	1	50	1:15	-0.873	-1.339
6	2	50	1:5	-0.206	-0.315
7	0.5	95	1:15	0.144	0.221
8	1	95	1:5	-0.606	-0.930
9	2	95	1:10	-0.863	-1.324
Mean 1	0.313	1.738	0.334		
Mean 2	0.364	-0.761	0.470		
Mean 3	-0.677	-0.978	-0.804		
Range (R)	1.041	2.716	1.274		

1.10 Analysis of Variance (ANOVA)

To statistically verify the significance of the factors affecting the comprehensive score, an Analysis of Variance (ANOVA) was conducted.

Table S5 Analysis of Variance (ANOVA) for the orthogonal test

Source of Variation	Sum of Squares (SS)	Degrees of Freedom (df)	F-value
Extraction Time	2.068	2	1.340

Ethanol Concentration	13.666	2	8.857*
Solid-Liquid Ratio	2.933	2	1.901

1.11 Verification of the Optimized Process

Based on the Range analysis (Table S3) and ANOVA (Table S5), Ethanol Concentration was the most significant factor. The optimal extraction conditions were determined as: 50% ethanol, solid-liquid ratio of 1:10, and hot maceration at 80°C for two cycles of 1 h each.

Verification experiments (n=3) under these conditions yielded a total active content of 72.18 ± 1.31 mg·mL^{-1} (RSD ≤ 3%), confirming the reproducibility and efficiency of the optimized process.

2 HPLC Method Validation

2.1 Specificity

The specificity of the method was assessed by comparing the chromatograms of the standard mixture and the APE-EP sample. As shown in Figure S1, the peaks of caffeic acid, chlorogenic acid, and cichoric acid were well-separated without interference from other components. Chromatographic analysis was performed on a YMC-Pack ODS-A C18 column (4.6 × 250 mm, 5 μm). The column temperature was maintained at 40 °C, and the detection wavelength was set at 332 nm. The mobile phase consisted of acetonitrile (solvent A) and 0.1% aqueous phosphoric acid (solvent B) with a flow rate of 1.5 mL/min. The injection volume was 10 μL. A gradient elution program was applied as follows: 0 – 13 min, 90% → 78% A; 13 – 14 min, 78% → 60% A; 14 – 20 min, held at 60% A.

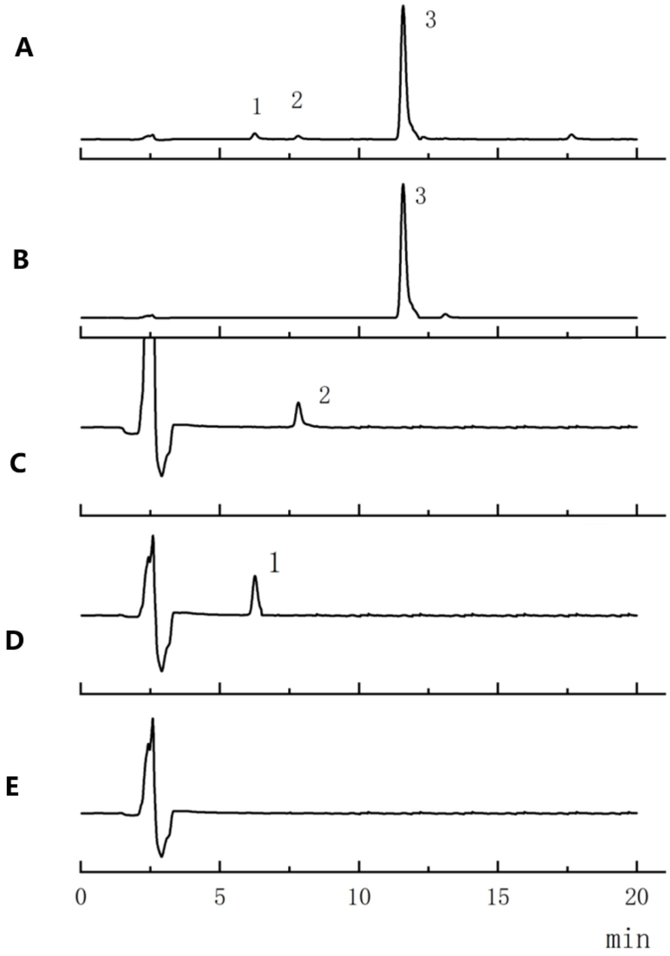

Figure S1 Chromatogram of HPLC. (A) Test sample solution; (B) Chicoric acid reference standard; (C) Chlorogenic acid reference standard; (D) Caffeic acid reference standard; (E) Blank solution. Peaks: 1, Caffeic acid; 2, Chlorogenic acid; 3, Chicoric acid. Note: Chromatograms are presented as stacked plots with offset baselines for visual clarity.

Table S6 Linear regression data, LOD, and LOQ of the three phenolic acids

Analyte	Regression Equation	R^2	Linear Range ($\mu g \cdot mL^{-1}$)	LOD (ng)	LOQ (ng)
Caffeic acid	Y = 229.16X + 447.27	0.9996	1.526 – 152.60	25.81	86.02
Chlorogenic acid	Y = 183.19X + 150.98	0.9995	0.710 – 72.10	22.51	74.96
Cichoric acid	Y = 2188.4X + 13421	0.9996	5.008 – 500.8	46.18	153.93

2.2 Precision

Precision was evaluated by intra-day and inter-day repeatability. Intra-day precision was determined by analyzing the sample solution five times within one day, while inter-day precision was determined over three consecutive days.

Table S7 Intra-day and inter-day precision of the HPLC method (n=5)

Analyte	Intra-day Precision		Inter-day Precision	
	Mean ± SD	RSD（%）	Mean ± SD	RSD（%）
Caffeic acid	152.69 ± 0.43	0.28	152.35 ± 1.77	1.16
Chlorogenic acid	71.97 ± 0.60	0.83	72.48 ± 1.86	2.56
Cichoric acid	251.68 ± 0.71	0.28	259.69 ± 0.47	0.18

2.3 Accuracy

The accuracy was verified by the spike recovery test. Known amounts of standards were added to the sample with known content, extracted, and analyzed (n=6).

Table S8 Recovery of the three phenolic acids (n=6)

Analyte	Sample Volume (mL)	Original Content (mg)	Added Amount (mg)	Found Amount (mg)	Recovery (%)	Average Recovery (%)	RSD (%)
Caffeic acid	2	66.876	79.120	141.057	96.617	96.62	1.41
	2	66.876	79.120	145.635	99.753		
	2	66.876	79.120	143.640	98.387		
	2	66.876	79.120	143.395	98.219		
	2	66.876	79.120	142.623	97.690		
	2	66.876	79.120	146.523	100.361		
Chlorogenic acid	2	34.832	40.975	73.205	96.567	96.57	4.86
	2	34.832	40.975	81.123	107.012		
	2	34.832	40.975	72.463	95.589		
	2	34.832	40.975	79.044	104.270		
	2	34.832	40.975	79.375	104.707		
	2	34.832	40.975	75.712	99.875		
Cichoric acid	2	117.287	107.958	225.910	100.295	100.30	1.25
	2	117.287	109.395	223.428	98.565		
	2	117.287	108.649	226.744	100.358		
	2	117.287	109.088	221.893	98.020		
	2	117.287	109.571	226.631	99.900		
	2	117.287	108.608	229.067	101.404		

Note: Recovery (%) = Found Amount / (Original Amount+Added Amount) × 100%.

3 Determination of Relative Correction Factors

The multi-point calibration method was adopted to determine the Relative Correction Factors (RCFs, $f_{s/i}$). The final fs/k value was obtained by calculating the average of the values derived from multiple mass concentration points. The calculation formula for the RCF is shown in Equation (2), and the formula for calculating the mass concentration of the analyte is shown in Equation (3). Additionally, the Relative Retention Time (RRT) for each analyte was calculated using the retention time of Caffeic acid as the reference.

$$f_{s/i} = \frac{A_s \times C_k}{A_k \times C_s} \qquad (2)$$

$$C_k = \frac{f_{s/i} \times C_a \times A_k}{A_s} \qquad (3)$$

Where A_s: Peak area of the Reference Substance (Caffeic acid).C_s: Mass concentration of the Reference Substance (Caffeic acid). A_k: Peak area of the analyte (Chlorogenic acid or Chicoric acid). C_k: Mass concentration of the analyte.

To determine the RCFs, 1, 2, 5, 10, and 20 μL of the mixed standard solution were precisely injected and analyzed. Caffeic acid was selected as the Reference Substance to calculate the RCFs for the other two components.

Robustness was evaluated by assessing the stability of RCFs under varying experimental conditions, specifically different injection volumes (representing different concentration levels) As presented in Table S9, the Relative Standard Deviations (RSDs) of the RCFs calculated from different injection volumes (1–20 μL) were all less than 1.5%. This high stability across a wide concentration range confirms that the RCFs are insensitive to sample load variations, thereby demonstrating the robustness and reliability of the QAMS method for routine analysis.

Table S9 Relative correction factors of components to be measured in samples

Sample Volume (μL)	Chlorogenic acid	Cichoric acid
1	0.215	7.945
2	0.212	7.977
5	0.213	7.950
10	0.219	7.920
20	0.215	7.912
Mean ± SD	0.215±0.003	7.941±0.026
RSD（%）	1.28	0.327

4 UPLC-Q-TOF-MS/MS Analysis

4.1 UPLC-Q-TOF-MS/MS Conditions

Chromatographic separation was performed on a Waters BEH C18 column (150 × 2.1 mm, 2.5 μm) maintained at 40 °C. The mobile phase consisted of acetonitrile (Solvent A) and 0.1% aqueous formic acid (Solvent B). The flow rate was set at 0.3 mL/min. A gradient

elution program was applied as follows: 0–2 min, 5% A; 2–17 min, 5% → 95% A; 17–18 min, 95% A; 18–19 min, 95% → 5% A; 19–21 min, 5% A.

Mass spectrometric detection was carried out using a TripleTOF system equipped with an electrospray ionization (ESI) source operating in both positive and negative ion modes. The optimized source parameters were set as follows: Ion Source Gas 1 (GS1) at 50 psi, Ion Source Gas 2 (GS2) at 50 psi, and Curtain Gas (CUR) at 25 psi. The source temperature was maintained at 500 °C for positive mode and 450 °C for negative mode. The Ion Spray Voltage Floating (ISVF) was set to 5500 V and −4400 V for positive and negative modes, respectively. Data acquisition was performed using Information Dependent Acquisition (IDA) in high-sensitivity mode. The TOF MS scan range was 100–1200 Da (accumulation time 0.2 s), while the product ion scan range was 50–1000 Da (accumulation time 0.01 s). The Declustering Potential (DP) was ±60 V, and the Collision Energy (CE) was set at 35 ± 15 eV.

4.2 Data Processing and Metabolite Identification

The raw data were converted to Analysis Base File (ABF) format using the Analysis Base File Converter. Subsequent data processing, including peak extraction, noise reduction, deconvolution, and alignment, was performed using MS-DIAL software (version 4.70). The extracted peaks were identified by matching their mass spectra and retention times against public databases, including MassBank, ReSpect, and GNPS. The detailed parameters for peak detection and identification were set as follows: minimum peak height, 1000 amplitude; mass slice width, 0.1 Da; retention time tolerance, 0.05 min; MS1 tolerance, 0.01 Da; MS2 tolerance, 0.05 Da; and identification score cutoff, 80. The typical total ion chromatograms (TIC) are shown in Supplementary Figure S2. The peak numbers correspond to the compound numbers listed inTable S10.

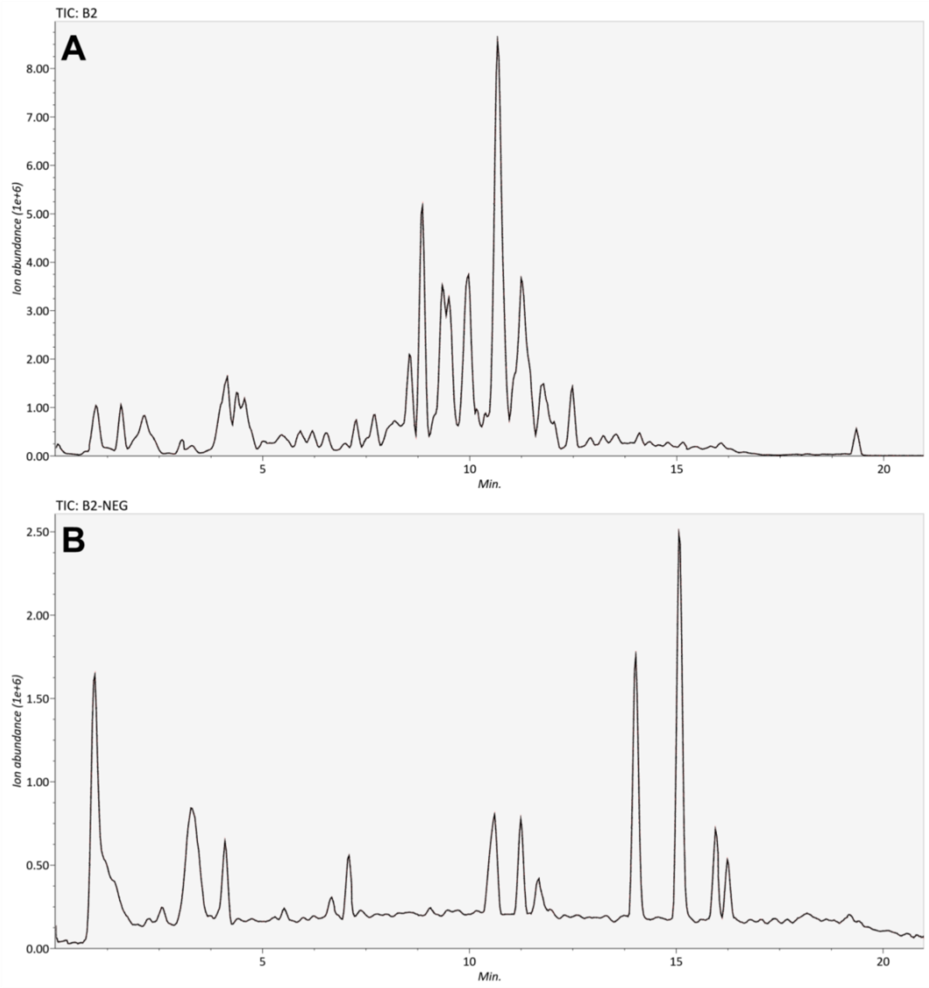

FigureS2 Representative UPLC-Q-TOF-MS/MS total ion chromatograms (TIC) of the aerial part extract of E. *purpurea*. (A) Positive ion mode; (B) Negative ion mode.

Table S10 LC-MS Analysis of Chemical Constituents in the aerial part extract of E. *purpurea*

No.	tR (min)	Precursor Ion (m/z)	Adduct	Formula	Compound Name	MS/MS Fragments (m/z)	Class
1	1.01	191.0561	[M−H]−	C7H12O6	Quinic acid	179	Organic acid
2	1.01	353.1	[M−H]−	C16H18O9	3-Caffeoylquinic acid	191, 179, 135	Organic acid
3	2.15	315.0709	[M−H]−	C13H16O9	Dihydroxybenzoic acid glucoside	153, 152, 109	Phenolic
4	2.37	269.078	[M−H]−	C15H10O5	Apigenin	251, 225, 151	Flavonoid
5	2.4	199.0601	[M+H]+	C9H10O5	Syringic acid	181, 147, 93	Organic acid
6	2.46	353.0862	[M−H]−	C16H18O9	Cryptochlorogenic acid	191, 173	Organic acid

7	2.76	342.17	[M+H]+	C20H23NO4	Magnoflorine	297, 265	Alkaloid
8	2.79	205.0975	[M+H]+	C11H12N2O2	Tryptophan	146	Amino acid
9	3.74	289.064	[M−H]−	C15H14O6	Catechin	245, 203, 161	Flavonoid
10	3.95	303.053	[M+H]+	C15H10O7	Quercetin	303	Flavonoid
11	4	609.1442	[M−H]−	C27H30O16	Rutin	301	Flavonoid
12	4.14	497.0722	[M+H]+	C22H18O12	Cichoric acid	335, 295	Organic acid
13	5.04	187.097	[M−H]−	C9H16O4	Azelaic acid	143, 125, 97	Organic acid
14	5.36	331.1358	[M−H]−	C17H16O7	3,4,2',4',5'-Pentahydroxy-6'-methoxyl-2-methylchalcone	169, 161, 125	Flavonoid
15	5.62	209.044	[M−H]−	C10H10O5	Hydroxyferulic acid	165, 121, 79	Organic acid
16	5.97	271.0823	[M−H]−	C12H16O7	Arbutin	108	Glycoside
17	7.98	367.1424	[M−H]−	C17H20O9	3-O-trans-Feruloylquinic acid	191, 173	Organic acid
18	6.29	431.096	[M−H]−	C21H20O10	Apigenin-4'-O-β-D-glucoside	269	Flavonoid
19	7.35	161.1058	[M−H]−	C8H8O4	Vanillic acid	123, 65	Organic acid
20	9.75	313.2375	[M−H]−	C18H34O4	Octadecanedioic acid	295, 201, 171	Fatty acid
21	11.24	295.2272	[M−H]−	C18H32O3	Hydroxylinoleic acid	171	Fatty acid
22	14.02	293.2121	[M−H]−	C18H30O3	Hydroxylinolenic acid	275, 235, 171	Fatty acid
23	15.84	257.2475	[M+H]+	C16H32O2	Palmitic acid	257	Fatty acid
24	13.9	271.2279	[M−H]−	C16H32O3	16-Hydroxypalmitic acid	195, 121	Fatty acid
25	15.06	279.2319	[M−H]−	C18H32O2	Linoleic acid	261, 205	Fatty acid
26	14.18	227.2009	[M−H]−	C14H28O2	Myristic acid	163, 101	Fatty acid
27	14.59	253.2171	[M−H]−	C16H30O2	Palmitoleic acid	253, 220	Fatty acid
28	16.23	281.2477	[M−H]−	C18H34O2	Oleic acid	281	Fatty acid
29	15.1	241.2204	[M−H]−	C15H30O2	Pentadecanoic acid	141, 123	Fatty acid
30	17.5	283.2617	[M−H]−	C18H36O2	Stearic acid	171, 166	Fatty acid
31	17.65	309.2793	[M−H]−	C20H38O2	Eicosenoic acid	309, 152	Fatty acid
32	18.98	311.293	[M−H]−	C20H40O2	Arachidic acid	311	Fatty acid
33	3.95	465.1025	[M+H]+	C21H20O12	Isoquercitrin	303, 167	Flavonoid
34	4.88	89.0599	[M+H]+	C4H8O2	Butyric acid	81, 56	Organic acid
35	9.94	316.2294	[M+H]+	C20H29NO2	Dodeca-2E,4E-dienoic acid 4-OH phenylethylamide	256, 179	Alkylamide
36	1.04	343.065	[M−H]−	C14H16O10	5-O-Galloylquinic acid	191, 169	Organic acid
37	1.8	271.0437	[M−H]−	C15H12O5	Naringenin	151, 107	Flavonoid
38	3.05	175.0599	[M−H]−	C7H12O5	Hydroxypimelic acid	131, 115	Organic acid

39	3.89	319.1283	[M+H]+	C15H10O8	Myricetin	130	Flavonoid
40	4	329.1211	[M−H]−	C17H14O7	Rhamnetin	271, 135	Flavonoid
41	5.46	409.2678	[M+H]+	C20H24O9	Nodakenin	128	Coumarin
42	6.58	195.1398	[M+H]+	C10H10O4	Caffeic acid methyl ester	163, 117	Phenylpropanoid
43	8.45	447.2081	[M−H]−	C21H20O11	Orientin	285, 153	Flavonoid
44	3.09	139.0415	[M+H]+	C7H6O3	p-Hydroxybenzoic acid	121, 95	Organic acid
45	8.95	257.1913	[M+H]+	C15H12O4	Liquiritigenin	137, 130	Flavonoid
46	9.49	449.2661	[M+H]+	C21H20O11	Quercitrin	303, 287	Flavonoid
47	10.33	285.2015	[M−H]−	C15H10O6	Luteolin	137, 119	Flavonoid
48	10.7	166.1226	[M+H]+	C9H11NO2	Phenylalanine	120, 67	Amino acid
49	0.92	299.0965	[M−H]−	C16H12O6	Kaempferol	284, 227	Flavonoid
50	1.92	204.1225	[M+H]+	C13H17NO	(2Z)-N-isobutyl-2-nonene-6,8-diynamide	130, 56	Alkylamide
51	2.18	224.0908	[M+H]+	C14H25NO	(2E,7Z)-N-isobutyl-2,7-decadienamide	131, 81	Alkylamide
52	4.81	248.167	[M+H]+	C16H25NO	(2E,4E,8Z,10Z)-N-isobutyl-dodeca-2,4,8,10-tetraenamide	220, 81	Alkylamide
53	5.88	278.1763	[M+H]+	C16H29NO	Undeca-2E,4E-diene-8,10-diynoic acid-2-phenylethylamide	135, 105	Alkylamide
54	7.61	336.1826	[M+H]+	C22H25NO2	Tetradeca-2E,4E-diene-8,10-diynoic acid 4-OH phenylethylamide	199, 121	Alkylamide
55	8.99	230.2477	[M+H]+	C15H19NO	(2E,4Z)-N-isobutyl-2,4-undecadiene-8,10-diynamide	102, 91	Alkylamide
56	9.88	268.1674	[M+H]+	C17H17NO2	N-phenethyl-2,3-epoxy-6,8-nonadiynamide	121, 91	Alkylamide
